# Molecular Diagnosis of Urinary Tract Infections by Semi-Quantitative Detection of Uropathogens in a Routine Clinical Hospital Setting

**DOI:** 10.1371/journal.pone.0150755

**Published:** 2016-03-08

**Authors:** Anneke van der Zee, Lieuwe Roorda, Gerda Bosman, Jacobus M. Ossewaarde

**Affiliations:** 1 Medical Microbiology, and Molecular Diagnostics Unit, Maasstad Hospital, Rotterdam, The Netherlands; 2 Erasmus Medical Centre, Rotterdam, The Netherlands; Naval Research Laboratory, UNITED STATES

## Abstract

**Background:**

The objective of our study was the development of a semi-quantitative real-time PCR to detect uropathogens. Two multiplex PCR reactions were designed to detect *Escherichia coli*, *Klebsiella spp*., *Enterobacter spp*., *Citrobacter spp*., *Proteus mirabilis*, *Enterococcus faecalis*, and *Pseudomonas aeruginosa*. 16S based PCR was performed in parallel to detect Gram-positive and Gram-negative bacteria. Firstly to identify non-targeted agents of infection in the same urine specimen, and secondly to quantify background flora. The method was evaluated in comparison with standard bacterial culture, and a commercial PCR kit for detection of uropathogens.

**Findings:**

Analysis with a known panel of 116 clinical isolates yielded a PCR specificity of 100%. Analysis of urine specimens from 211 patients revealed a high correlation of PCR Cq values with both culture positivity and quantity. Concordance between PCR and culture was 98% when both methods yielded results. PCR was found to be more sensitive than culture. With a cut-off Cq value of 33, the negative predictive value of PCR was 94%. The 16S PCR confirmed most results. One specimen was positive by 16S PCR suggesting another cause of infection not detected by the specific PCR assays.

**Conclusion:**

We conclude that it is feasible to detect and identify uropathogens by multiplex real-time PCR assay.

## Introduction

Acute uncomplicated urinary tract infections (UTI) are one of the most common bacterial infections, and have a high tendency to recur [1, 2]. A national survey in The Netherlands showed that UTIs were the most common healthcare associated infections [3]. UTIs are often caused by uropathogens normally residing in the intestines or the genital tract [4]. Virulence factors expressed by these organisms allow for their adherence to the mucosa of the urethra.

UTI can be difficult to diagnose. Often, patients are treated on symptoms alone [5], but the diagnosis of UTI by clinical criteria alone has an error rate of approximately 33% [6]. Conventional diagnosis is carried out by microscopy and culture to confirm the presence of leucocytes and bacteria in urine culture respectively and takes at least two days. However, bacteriuria can also occur in asymptomatic persons, depending on the population. The prevalence of bacteria in the urine may vary from 2% in young females to 10% in elderly asymptomatic women [6]. In addition, specimens may be contaminated with mucosal or fecal flora, especially in females. The ‘gold standard’ for diagnosis of UTI is culture based identification and quantification of organisms. According to the Infectious Disease Society of America, the definition for evidence of UTI requires growth of 10^3^ organisms per ml to diagnose cystitis and 10^5^ per ml for pyelonephritis [7].

It has been shown that bacteria causing UTI are increasingly resistant to antibiotics [8]. Because of the risk of increasing antimicrobial resistance, it is important to treat only patients with laboratory based evidence of UTI. A study conducted in general practices showed that 27% of patients were treated in the absence of significant bacteriuria. The report however did not confirm that antibiotic resistance of uropathogens had increased over a 10-year period [9], by contrast to the findings reported by Wagenlehner et al. [8]. In a hospital associated patient population, like the one in our study, where resistance to antibiotics occurs frequently, evidence based treatment may even be more important [10].

Molecular based diagnosis has the advantage that it can identify the cause of UTI within hours. Real-time PCR is increasingly used to diagnose systemic blood infections [11], or genital infections [12]. Molecular based methods for diagnosis of UTI have been described previously [13, 14]. Some are limited to detection of a single pathogen [15, 16], or detect only Gram positive or negative bacteria [17, 18]. Lehman et al. [19] described a 16S based real-time PCR using a large number of genus/species specific probes.

Here, we describe the development and evaluation of two real-time multiplex PCRs for detection of nearly all clinically relevant uropathogens. Both PCRs target single copy genes to allow for identification and semi quantification of bacteria present in urine. A 16S based PCR for detection of gram-positive, and gram-negative flora was used to identify other causative micro-organisms of UTI, and to measure common background flora in urine specimens.

## Results

### Analytical performance of PCRs

**Fig 1:**
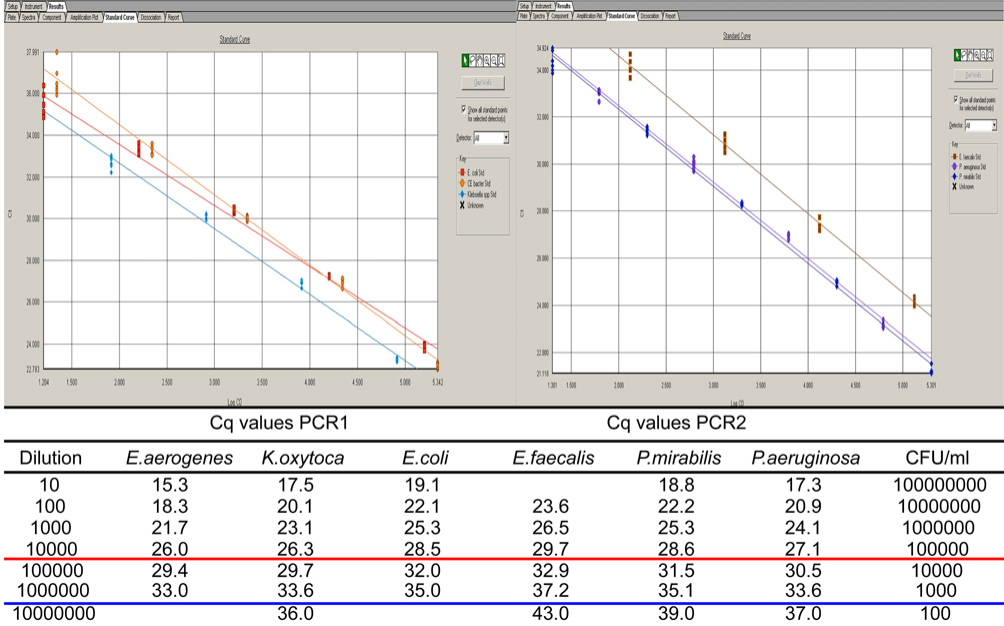
Amplification of serial dilutions of bacterial cells. Standard curves (top) of PCRs for detection of *Citrobacter/Enterobacter spp*. (yellow), *Klebsiella spp*. (blue), *E*.*coli* (red), *E*.*faecalis* (brown), *P*.*mirabilis* (dark blue), and *P*.*aeruginosa* (purple). The Cq values per dilution are shown below the figure. Cq values that correspond with 10^3^ and 10^5^ cfu/ml were underlined in blue and red, respectively.

For comparative quantification of potential uropathogens, single gene targets were selected for PCR. Their reliability, i.e. the widespread presence of the selected genes and their homology with regard to primer matching was assessed by amplification of 6 clinical strains from different patients for each PCR. Only when all strains were positive and amplification was efficient, the target genes were selected (S1 Table). To ensure good coverage of detection of all species within a genus, in some cases more than 1 target gene was selected. For example, a highly specific unnamed sequence (Xx in S1 Table) appeared to be present only in *Enterobacter cloaca*. For *E*. *aerogenes* Omp35 was chosen as target. Likewise, we found no shared gene between *K*. *pneumoniae* and *K*. *oxytoca*. (S1 Table. Primers and probes used for detection of target genes of uropathogenic bacteria). The quantification by single target amplification in serial dilutions of uropathogens is demonstrated in Fig 1.

A quantity of 10^3^ colony forming units (CFU) per ml can be detected in all cases with an approximate Cq value of 33. The multiplex combinations were made with stringent requirement that no loss of sensitivity occurred compared to monoplex PCR reactions. Using DNA mixtures of 3 different strains, which differed <100 in concentration, we investigated whether amplification of each separate strain was consistent with amplification in single strain DNA solutions. All required criteria were met with multiplex PCR combinations as described in Materials and Methods (not shown). The specificity of target genes was 100% in a panel of 116 clinical isolates comprising 28 different (sub)species (S2 Table. Results of PCRs on different clinical cultures).

### Results of PCR and culture

Urine specimens from 211 patients were analyzed in parallel by culture and PCR. A cut-off value of Cq 33 was applied to PCR since this value sets the limit for approximately 10^3^ bacteria per ml (Fig 1).

Of 211 patients, 86 patients were negative both by culture (CFU<10^3^), and by PCR (Cq>33).

Sixty-two patients were positive in PCR and 44 of these patients had a positive culture (Table 1). Eighteen patients were PCR positive but no significant culture result could be obtained (Table 2). Ten patients had positive cultures which could not be confirmed by species specific PCR. Some could only weakly be supported by 16S based PCR (Table 3), while another 53 patients had inconclusive results both in PCR and culture (Table 3).

**Table 1:** PCR positive and culture positive urine specimens (n = 44).

No	PCR positive for organism(s):			Culture positive for organism(s):		Quantitycfu/ml
21	*E*.*coli*			*E*. *coli*		10^4^–>10^5^
1	*E*.*coli*	*E*. *faecalis*		*E*. *coli*		10^4^
1	*E*. *coli*	*E*. *faecalis*		*Streptococcus* spp.		10^4^
1	*E*. *faecalis*			*E*. *faecalis*	*E*. *coli*	10^3^/10^3^
3	*P*. *mirabilis*			*P*. *mirabilis*		>10^5^
1	*P*. *mirabilis*	*E*. *coli*		*P*. *mirabilis*	*E*. *coli*	10^4^/10^4^
1	*P*. *mirabilis*	*E*. *coli*		*P*. *mirabilis*		>10^5^
1	*P*. *mirabilis*	*P*. *aeruginosa*	CEbacter*	*P*. *mirabilis*	*S*. *aureus*	>10^5^/nd
3	*P*. *aeruginosa*			*P*. *aeruginosa*		10^4^
1	*P*. *aeruginosa*	Klebsiella spp.		*P*. *aeruginosa*	*K*. *pneumoniae*	10^4^/>10^5^
4	*Klebsiella* spp.			*K*. *pneumoniae*		10^3^–>10^5^
1	*Klebsiella* spp.	*E*. *coli*	*E*. *faecalis*	*E*. *coli*		>10^5^
1	*Klebsiella* spp.			*K*. *oxytoca*	*Streptococcus* spp.	>10^5^/nd
1	*Klebsiella* spp.	*E*. *faecalis*		*K*. *pneumoniae*		10^4^
1	CEbacter*			*E*. *cloacae*		>10^5^
1	CEbacter*			*E*. *cloacae*	*P*. *mirabilis*	10^3^/10^3^
1	CEbacter*	*E*. *faecalis*		*C*. *koseri*		>10^5^

*Citrobacter spp. and/or Enterobacter spp.

nd, not determined

Quantities apply only to cultures

**Table 2:** PCR positive urine specimens with inconclusive culture results (n = 18).

No	PCR		Culture	Quantity cfu/ml
*4*	*E*. *coli*		mixed flora, possible contamination	<10^3^–>10^5^
*1*	*E*. *coli*	*P*.*mirabilis*	mixed flora, possible contamination	>10^5^
*1*	*E*. *coli*	*E*. *faecalis*	mixed flora, possible contamination	>10^5^
*7*	*E*. *faecalis*		negative/mixed flora, possible contamination	<10^3^–>10^5^
*1*	*P*. *aeruginosa*		negative	<10^3^
*1*	*P*. *aeruginosa*	*E*. *faecalis*	mixed flora, possible contamination	<10^3^
*1*	CEbacter*		negative	<10^3^
*1*	*Klebsiella* spp.		mixed flora, possible contamination	>10^5^
*1*	*Klebsiella* spp.	*P*. *mirabilis*	mixed flora, possible contamination	>10^5^

*Citrobacter spp. and/or *Enterobacter* spp.

Quantities apply only to cultures

**Table 3:** Results of 16S PCR for Gram-positive and Gram-negative organisms with positive culture results (n = 10) and inconclusive culture results (n = 53) and negative in species specific PCRs.

No	Cq values Gram-positive PCR	Cq values Gram-negative PCR	Culture	Quantity cfu/ml
1	31,1	24,2	*E*. *coli*	>10^5^
1	>50	39,7	*E*. *coli*	10^3^
1	37,2	>50	*E*. *coli*	10^3^
1	26,8	>50	*E*. *coli*	10^3^
3	28,4	>50	*Streptococcus* spp.	>10^5^
1	28,6	>50	*A*. *lwoffii*	10^4^
1	45,6	34,6	*S*. *aureus*	10^3^
1	29,3	>50	*Candida glabrata*	nd
5	21.3–38.4	>50	mixed flora, possible contamination	10^3^->10^5^
8	27.3–38.2	>50	negative	<10^3^
20	27.3–39.4	>50	mixed flora, possible contamination	10^4^
7	32.2–36.2	>50	negative	<10^3^
2	>50	29.4–32.5	negative	<10^3^
2	>50	32.1–32.6	mucosal flora,possible contamination	10^3^
1	>50	25.0	mixed flora, possible contamination	10^4^
1	42.8	32.0	negative	<10^3^
5	31.5-	39.0-	mucosal flora,possible contamination	10^3^−10^4^
2	36.5–38.3	30.7–34.8	mixed flora, possible contamination	10^4^

**Fig 2:**
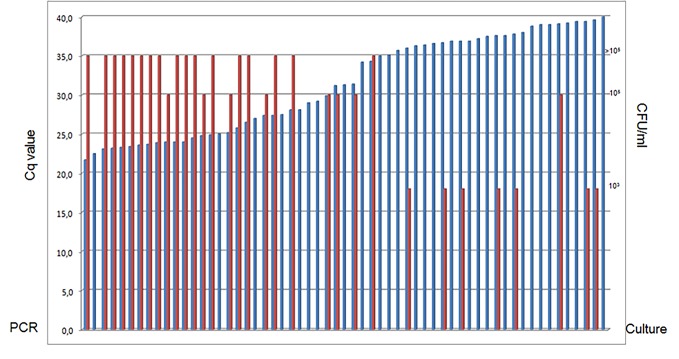
Graphic showing the correlation between positive cultures and PCR Cq values. Comparison of PCR and culture results for *E*. *coli*. Cq values of PCR are depicted in the blue bars ranging from Cq22-Cq40 on the left axis. The red bars depict positive cultures with growth of 10^3^, 10^5^, and >10^5^ cfu/ml on the right axis. Low Cq values correspond with high loads of micro-organisms, and thus with larger yield of positivity and quantities of cultures.

Of 44 PCR positive specimens, 2 specimens were positive for 3 uropathogens but only one was confirmed by culture. Seven specimens were PCR positive for 2 uropathogens, 4 of which were culture positive for one, 2 were culture confirmed for both, and one specimen was culture negative for the PCR detected pathogens but positive for *Streptococcus* spp. which is not detected by PCRs. PCR detected single pathogens in the remaining 35 specimens, and all were confirmed by culture. In 5 cases an additional potential pathogen was cultured (Table 1). Of 18 PCR positive specimens, 4 were positive for 2 pathogens, but culture results were inconclusive (Table 2). The mean Cq value of these PCRs was 29.0, and the mean Cq value of culture confirmed PCRs was 26.9. In general, the quantity of growth (CFU per ml) decreased with increasing Cq value, which is shown for *E*. *coli* in Fig 2. Among all specimens, the highest rate of culture positivity was found when Cq values were lowest, and hence bacterial loads were high. One specimen showed inhibition of PCR. The concordance of PCR and culture is 40% due to the higher sensitivity of PCR. The concordance is 98% (43/44), when culture results are available. Statistical analysis showed no significant difference between the outcomes of PCR and culture (Table 4, p = 0. 1859).

**Table 4:** Analysis of results of PCR and culture.

	*PCR		
*Culture	Positive	Negative	Total
Positive	44	10	54
Negative	18	86	104
Total	62	96	158
Overall agreement = 82%			
p = 0.1859			
		Culture	PCR
Prevalence		34%	39%
Sensitivity		71%	81%
Specificity		83%	90%

*53 patients with inconclusive results were left out

### Evaluation of 16S PCR for support and detection of other potential uropathogens

The 16S PCR for detection of Gram-positive and Gram-negative bacteria was used in parallel with specific PCR’s. This was done to investigate its usefulness in detection of non-targeted uropathogens, and of background of common mucosal or fecal flora. For 16S PCR a cut-off Cq value of 30 was set because all cultures were negative with 16S PCR Cq values >30. Cq values of 16S PCR are lower compared to specific PCRs, which can be explained by the fact that the 16S target gene may be present in multiple copies, but also because common background flora is detected.

Nearly all PCRs for detection of specific organisms were confirmed by 16S based PCR for detection of Gram-positive (*E*. *faecalis*), and Gram-negative bacteria. The exceptions were: one PCR positive for *P*. *mirabilis* where Gram-positive 16S PCR was positive with Cq 23.6 for background mucosal flora; four PCR positive for *E*. *faecalis* where Gram-negative 16S PCR was positive; and four PCR positive for *P*. *aeruginosa* also were not confirmed by 16S PCR. It should be noted that the *P*. *aeruginosa* 16S gene does not have a perfect match with the probe which results in higher Cq values compared to other uropathogens.

Of 10 culture positive specimens (Table 3) that were negative in species specific PCRs, only one *E*. *coli* could be confirmed by 16S PCR with Cq 24.2, and one *Streptococcus* spp. with Cq 28.4, while other cultures were negative in 16S PCR. Among specimens with inconclusive culture results Cq values for Gram-negative bacteria are high (>29.4) and compatible with background flora. Cq values for Gram-positive bacteria were >22 with a mean Cq value of 27.6, indicating that positivity most likely was derived from common mucosal flora. Positive PCRs for Gram-positive organisms occurred more often in specimens obtained from females (80%). The species specific PCRs lack the ability to detect the following organisms that were found with culture; *Staphylococcus aureus*, *Streptococcus spp*., *Acinetobacter lwolfii*, and *Candida glabrata*. Except for *Candida spp*., 16S PCR can be expected to detect all Gram-positive and Gram-negative organisms, and therefore other possible causes of UTI. When species specific PCRs were judged to be negative (Cq>33), 16S PCR was positive (Cq<30) in 23 of the remaining 137 specimens. In the Gram-negative 16S based PCR, two were positive, and one of 137 specimens had a Cq value of 25.0 (Table 3), which suggests another Gram-negative bacteria as a possible cause of UTI. However, the culture positive Gram-negative *Acinetobacter lwoffii* was not detected.

### Comparison of PCR and the Seegene kit

In a subgroup of 83 patients results of our PCR assays were compared to the Seeplex UTI ACE Detection kit, which employs conventional PCR (non-quantitative), and electrophoresis of amplified products. The results of the comparison are shown in Table 5. The detection of *Citrobacter spp*. and *Enterobacter spp*. was omitted because they are not detected using the Seegene kit. *S*. *saprophyticus* was not detected at all, and was omitted as well. The concordance between the two methods is high (Table 6, p = 0.0433). Most notable is that of all PCR positive *E*. *faecalis*, all were also positive with Seegene detection. Of 47 Seegene negatives, 12 were culture positive for *E*. *coli*, one for *Enterococcus spp*., one for *K*. *pneumoniae*, and one *P*. *mirabilis*. In four specimens inhibition of PCR occurred. All Seegene positives were also identified with real-time multiplex PCRs. Of all Seegene positive specimens 48% (15/31) were concordant with the results of the culture.

**Table 5:** Comparison of results of multiplex PCR and Seegene (n = 83)

Detector	Seegene	PCR	Agreements	Discrepancies	organisms
*E*. *coli*	14	11	11	3	2 *K*. *pneumoniae*, 1 *P*. *mirabilis*
*E*. *faecalis*	10	10	10	0	
*K*. *pneumoniae*	5	7	5	2	2 *Klebsiella spp*.
*P*. *mirabilis*	4	3	3	1	1 CEbacter
*P*. *aeruginosa*	3	3	3	0	
negative	47	37	37	10	6 *E*. *coli*, 2 *Klebsiella spp*., 1 *P*. *aeruginosa*, 1 *P*. *mirabilis*
inhibition	4	1	1	3	

**Table 6:** Analysis of results of PCR and the Seegene kit.

	PCR		
Seegene	Positive	Negative	Total
Positive	34	2	36
Negative	10	37	47
Total	44	39	83

Overall agreement = 86%

p = 0.0433

## Discussion

To improve the laboratory diagnosis of UTI, we developed a real-time PCR assay to detect the most common causes of UTI. Using single gene targets the PCR described here allows quantification of identified uropathogens. Single gene targets were selected with care and consisted of genes associated with virulence, housekeeping, or encoding outer membrane proteins. *E*. *coli* is most frequently found associated with UTI. For amplification of this uropathogen we chose a virulence associated gene as target in PCR. The RfaH gene encodes a transcriptional regulator for fimbriae expression. Since fimbriae are regarded as an absolute requirement for adherence and infection of the urethra [20], the gene may be expected to be present in all uropathogenic *E*. *coli*. Nevertheless, during our evaluation *E*. *coli* was 8 times cultured, while PCR was judged negative when applying a cut-off of Cq 33. Also Gram-negative PCR provided no proof of infection in 7 *E*. *coli* culture positive specimens. Isolation of uropathogens is regarded the ‘gold standard’. However, differences in growth rates, and the requirement for specific media may confound culture results. Specific pathogens may be overgrown by common flora or not grow at all. The apparent insensitivity of culture for growth of *E*. *faecalis* is notable. Only one specimen was culture positive whereas PCR (Cq<33) identified *E*. *faecalis* in 14 urine specimens. All but two were also confirmed to be positive by the commercial Seegene kit for detection of uropathogens.

Real-time PCR compared well to detection by the Seegene kit, and showed a slightly higher sensitivity of detection. The advantage of real-time PCR identification of uropathogens is that results are available within a few hours. Seegene based detection is more laborious and time-consuming since PCR products need to be analyzed by gel electrophoresis.

We have shown that PCR based detection can replace culture based diagnosis, provided that no antibiotic sensitivity testing is required for adequate treatment of patients. Real-time PCR is by definition at least semi-quantitative. Low Ct values are indicative of high loads and vice versa. Having made standard curves of the relation between loads and Ct values, quantification can be done with higher precision. Hence instead of Ct, Cq is used. With PCR, safely applying a cut-off value of Cq 40, at least 42% (88) of culturing can be eliminated. The negative predictive value of PCR in this case is 100%. With a cut-off Cq value of 33, the negative predictive value of PCR would be 94% mainly due to growth of *E*. *coli*, and *P*. *mirabilis* from specimens with high or negative Cq values in PCR.

The 16S PCR confirmed most results. Only one specimen was found with Gram-negative PCR which was suspect to contain another cause for infection. The correlation of specific PCR and 16S was very high. Only when Cq values of either Gram-positive or Gram-negative PCR were low (Cq<25), competition for reaction components appeared to hamper reliable detection of both, a finding which has been presented before [19]. This drawback can be overcome by separation of Gram-positive and Gram-negative PCR into two reactions.

The limitation of our study may be that we have not been able to test enough strains to ensure reliable presence and homology of target genes. This requires further study. The PCR detection can be extended to detection of additional targets such as *Acinetobacter spp*. or *Candida spp*. Nevertheless, we have shown that real-time PCR based diagnosis is feasible in the identification and determination of the most frequently found causes of urinary tract infections.

## Materials & Methods

### Ethics Statement

After being used for culture, urine specimens are discarded. For this study we used waste material. Since no extra action or sampling was requested other than the medically indicated specimen collection, we did not ask for informed consent. Patient data was analyzed anonymously. Our institutional review board (Toetsingscommissie Wetenschappelijk Onderzoek Rotterdam e.o.) waived the need for written informed consent from the participants and approved of the study under L201585, in agreement with national law by the Federation of Dutch Medical Scientific Societies (www.Federa.org).

### Patients and specimens

Urine specimens were used from 211 hospitalized or outpatients; 57% were female, mean age 54 years, range 2–96 years, median 56, and 43% were male, mean age 60 years, range 1–88 years, median 62. Patients were from various departments, 5 patients were in ICU, 12 patients had a catheter. All patients were suspected for having UTI.

### Culture

One μl of urine specimen was cultured on blood agar and McConkey agar (Oxoid). The presence of 10^3^ colony-forming units (CFUs) of a single uropathogen or more per milliliter was considered positive. Cultures were quantified from 10^3^, 10^4^ to >10^5^. Identification and antibiotic susceptibility testing was performed according to standard microbiological procedures.

### PCR

Prior to PCR, DNA from urine specimens was extracted using Sigma extraction buffer (E7526, Sigma-Aldrich, Munich, Germany). In short, 50 μl of urine was added to 100 μl Extraction Solution, heated for 10 min. at 95°C, and supplemented with 100 μl Dilution Buffer (D5688, Sigma). In PCR, 5 μl of template was added, which corresponds to the equivalent of 1 μl of unprocessed urine. PCRs were designed based on single copy genes which allowed for semi-quantitative detection of one pathogen, genus or group (S1 Table). PCR mixture 1 contained primers and probes for detection of *E*. *coli*, *Citrobacter* spp. and *Enterobacter* spp., and *Klebsiella* spp. PCR mixture 2 was for detection of *P*. *mirabilis*, *E*. *faecalis*, and *P*. *aeruginosa*. PCR mixture 3 contained primers and probes based on the 16S gene and designed to detect and discriminate Gram-negative and Gram-positive bacteria (S2 Table). All multiplex reactions were internally controlled by addition of Phocine Herpes virus. PCRs were performed in a Reaction Mix (E3004, Sigma Aldrich, Germany) of 25 μl. Amplification was carried out on an ABI 7500 Real-Time PCR system (Applied Biosystems (ABI), Life Tech, Glasgow, UK). The temperature profile included initial denaturation of 4 min. at 94°C, followed by 50 cycles of 94°C for 15 sec., and 60°C for 1 min. Cycle treshold (Cq) values were determined automatically using the ABI SDS software.

### Evaluation of performance of PCRs

The analytical evaluation of single target detection was assessed by investigation of PCR performance with regard to Cq values for (semi)-quantification, compatibility of combinations, fidelity of amplification, and specificity. The specificity of PCRs was investigated using 116 clinical isolates comprising 28 (sub)species (S2 Table. Results of PCRs on different clinical cultures).

For evaluation of multiplex PCRs results were compared with the outcome of culture in a group of 211 patients, and with Seegene, Seeplex UTI ACE detection (Seegene, Eschborn, Germany) of uropathogenic *E*. *coli*, *Proteus mirabilis*, *Klebsiella pneumonia*, *Staphylococcus saprophyticus*, *Pseudomonas aeruginosa*, and *Enterococcus faecalis* in a group of 83 patients. PCR products of the UTI ACE Detection were analysed using the MultiNA (MCE^®^-202, Shimadzu, Korea).

### Statistical analysis

McNemar’s Chi-squared test to check for equality in the absence of a reference standard (GraphPad Prism 6) was used to compare outcomes of real-time PCR, and culture, and PCR, and the commercial kit of Seegene for detection of uropathogens.

## Supporting Information

S1 Table Primers and probes used for detection of target genes of uropathogenic bacteria. (DOCX)

S2 Table Results of PCRs on different clinical cultures. (DOCX)
