# Supplementary material for: Molecular Diagnosis of Urinary Tract Infections by Semi-Quantitative Detection of Uropathogens in a Routine Clinical Hospital Setting
Source: PLoS One. 2016 Mar 8;11(3):e0150755. doi: 10.1371/journal.pone.0150755 (PMC4783162; doi:10.1371/journal.pone.0150755)
Supplement: S1 Table — (DOCX) [file pone.0150755.s001.docx]

S1 Table.

Primers and probes used for detection of target genes of uropathogenic bacteria.

| Organism | target |  | oligonucleotides (5'-3') | reference |
| --- | --- | --- | --- | --- |
| *E. coli* | RfaH | F | TACGCCCGCCGTTGAC | This study |
|  | RfaH | R | AGCCAGCAGGCGCAAA |  |
|  | RfaH | CY5 | AACAGGACGAATACTGACGCGCCA |  |
| *Klebsiella spp.* | CopG | F | CGAAGAAGACGGCATGGAAT | This study |
|  | CopG | R | CGCAGATCCGGAGGTCATTA |  |
|  | CopG | YY | TCAACGTCAGCTACGCAAGGAGTG |  |
|  | Pgi | F3 | GAAGGTGAAGATGTTCATAATCACG | This study |
|  | Pgi | R2 | CGTGAAATCACGCCGTTCAG |  |
|  | Pgi | R3 | GCGTGAAATTAAGCCGTTCAG |  |
|  | Pgi | YY | CATACAGGGCAATCAGCGCGCC |  |
| *Citrobacter spp.* | MrkC | F1 | ATGTGTATACCAATGGTGAGTGGAA | This study |
|  | MrkC | R1 | GCCCATTTTGCCGTCTTTT |  |
|  | MrkC | FAM | CCGCTACGACCTGAATATCACCCGTG |  |
|  | Cfa | F1 | CCTGGTGGCCGGGATT | Ref. (9) |
|  | Cfa | R1 | GTCCAGCGCATTCAGATGAGT |  |
|  | Cfa | R3 | GGCATCAAGCGCATTAAGATGTAT |  |
|  | Cfa | FAM | CCGCAACCCTTTCCGATCTGGAG |  |
| *Enterobacter spp.* | Xx | F1 | ACAAAGGAGTCGGGATGAGTTC | This study |
|  | Xx | R1 | CGACCATTGCTCGTAAGGCT |  |
|  | Xx | FAM | CAATCCCAGGCCAAATCACCGG |  |
|  | Omp | F2 | CCCATGCTTCAGCTTTGTCA | This study |
|  | Omp | R2 | CTGCAGGTTACGCTAACTCCAA |  |
|  | Omp | FAM | CGTTGCCGTCACGTTTCTGGTCAA |  |
| *P. aeruginosa* | OprL | F1 | GCGTCGAGCTGAAGAAGTAAGAAG | This study |
|  | OprL | R | GGCTGAGCGCGAGGAA |  |
|  | OprL | CY5 | CCCAAGCACCTGCGTGTCCTGAC |  |
| *E. faecalis* | RecG | F2 | GCGAAATGGATGTATCAATCATTG | This study |
|  | RecG | R | TTTCCATCCATTCTAAAACAGTATCTAACT |  |
|  | RecG | YY | ACACGTTGGATTCGGCCGCC |  |
| *P. mirabilis* | Hns | F | GCACGTTTAGCACGACCAGTT | This study |
|  | Hns | R | TGCCGATGGTATTGATCCAA |  |
|  | Hns | FAM | CGCCAGCAGCTTCAAGCAGGTCA |  |
| Gram-negative | 16S | F2 | ACTCCTACGGGAGGCAGCAGT | Ref. (10) |
|  | 16S | R2 | ACGTATTACCGCGGCTGCT |  |
|  | 16S | YY | CCGCAGAATAAGCACCGGCTAACTCCGT |  |
| Gram-positive | 16S | F1 | GCAGCAAACGCATTAAGCACT | This study |
|  | 16S | R1 | GTTCTTCGCGTTGCTTCGA |  |
|  | 16S | FAM | CGACCGCAAGGTTGAAACTCAAAGGA |  |
|  | 16S | F3 | AGTGACAGGTGGTGCATGGTT |  |
|  | 16S | R3 | CATAAGGGGCATGATGATTTGA |  |
|  | 16S | FAM | TCGTCAGCTCGTGTCGTGAGATGTTG |  |
