# Supplementary material for: Molecular Diagnosis of Urinary Tract Infections by Semi-Quantitative Detection of Uropathogens in a Routine Clinical Hospital Setting
Source: PLoS One. 2016 Mar 8;11(3):e0150755. doi: 10.1371/journal.pone.0150755 (PMC4783162; doi:10.1371/journal.pone.0150755)
Supplement: S2 Table — (DOCX) [file pone.0150755.s002.docx]

S2 Table.

Results of PCRs on different clinical cultures.

| No | Organism | PCR |
| --- | --- | --- |
| 9 | *Escherichia coli* | positive |
| 6 | *Shigella spp.* | positive |
| 9 | *Klebsiella pneumoniae* | positive |
| 5 | *Klebsiella oxytoca* | positive |
| 3 | *Enterobacter aerogenes* | positive |
| 5 | *Enterobacter cloacae* | positive |
| 3 | *Citrobacter freundii* | positive |
| 5 | *Citrobacter braakii* | positive |
| 8 | *Citrobacter koseri* | positive |
| 5 | *Proteus mirabilis* | positive |
| 9 | *Enterococcus faecalis* | positive |
| 5 | *Pseudomonas aeruginosa* | positive |
| 1 | *Citrobacter dipht gravidi* | negative |
| 1 | *Citrobacter dipht mitis* | negative |
| 1 | *Citrobacter ulcerans* | negative |
| 1 | *Citrobacter stratium* | negative |
| 1 | *Citrobacter propiquum* | negative |
| 3 | *Proteus vulgaris* | negative |
| 6 | *Enterococcus faecium* | negative |
| 1 | *Corynebacterium spp.* | negative |
| 1 | *Rhizobium radiobacter* | negative |
| 6 | *Campylobacter jejuni* | negative |
| 1 | *Campylobacter coli* | negative |
| 2 | *Campylobacter lari* | negative |
| 5 | *Staphylococcus spp.* | negative |
| 5 | *Staphylococcus aureus* | negative |
| 4 | *Staphylococcus saprophyticus* | negative |
| 5 | *Streptococcus spp.* | negative |
